# SCN5A Nonsense Mutation and NF1 Frameshift Mutation in a Family With Brugada Syndrome and Neurofibromatosis

**DOI:** 10.3389/fgene.2019.00050

**Published:** 2019-02-15

**Authors:** Emanuele Micaglio, Michelle M. Monasky, Giuseppe Ciconte, Gabriele Vicedomini, Manuel Conti, Valerio Mecarocci, Luigi Giannelli, Federica Giordano, Alberto Pollina, Massimo Saviano, Simonetta Crisà, Valeria Borrelli, Andrea Ghiroldi, Sara D’Imperio, Chiara Di Resta, Sara Benedetti, Maurizio Ferrari, Vincenzo Santinelli, Luigi Anastasia, Carlo Pappone

**Affiliations:** ^1^ Arrhythmology Department, IRCCS Policlinico San Donato, San Donato Milanese, Italy; ^2^ Stem Cells for Tissue Engineering Laboratory, IRCCS Policlinico San Donato, San Donato Milanese, Italy; ^3^ Genomic Unit for the Diagnosis of Human Pathologies, Division of Genetics and Cellular Biology, IRCCS San Raffaele Hospital, Milan, Italy; ^4^ Vita-Salute San Raffaele University, Milan, Italy; ^5^ Laboratory of Clinical Molecular Biology and Cytogenetics, IRCCS San Raffaele Hospital, Milan, Italy; ^6^ Department of Biomedical Sciences for Health, University of Milan, Milan, Italy

**Keywords:** Brugada syndrome, neurofibromatosis type 1, sudden cardiac death, genetic testing, mutation, arrhythmia, SCN5A, NF1

## Abstract

In this case series, we report for the first time a family in which the inherited nonsense mutation [c. 3946C > T (p.Arg1316*)] in the *SCN5A* gene segregates in association with Brugada syndrome (BrS). Moreover, we also report, for the first time, the frameshift mutation [c.7686delG (p.Ile2563fsX40)] in the *NF1* gene, as well as its association with type 1 neurofibromatosis (NF1), characterized by pigmentary lesions (café au lait spots, Lisch nodules, freckling) and cutaneous neurofibromas. Both of these mutations and associated phenotypes were discovered in the same family. This genetic association may identify a subset of patients at higher risk of sudden cardiac death who require the appropriate electrophysiological evaluation. This case series highlights the importance of genetic testing not only to molecularly confirm the pathology but also to identify asymptomatic family members who need clinical examinations and preventive interventions, as well as to advise about the possibility of avoiding recurrence risk with medically assisted reproduction.

## Background

The Brugada syndrome (BrS) is an autosomal dominant condition with extreme clinical variability and incomplete penetrance (Nademanee et al., 2011; Lieve and Wilde, 2015; Monasky et al., 2018). Brugada syndrome is characterized by a coved-type ST-segment elevation in the right precordial leads on the electrocardiogram (ECG) and by an increased risk of sudden cardiac death (SCD) (Antzelevitch et al., 2016). Patients with a spontaneous type 1 BrS ECG pattern are considered at higher risk for SCD, although patients can also be diagnosed by administration of a sodium channel blocking agent, such as ajmaline (Antzelevitch et al., 2016), which reveals the type 1 pattern. Patients can benefit from catheter ablation of the arrhythmogenic substrate (AS) located in the epicardial surface of the right ventricle (RV) (Nademanee et al., 2011; Zhang et al., 2015), preventing ventricular arrhythmias (VA) recurrences. The administration of ajmaline during the epicardial catheter ablation procedure helps to determine the exact location and full extent of the AS (Pappone et al., 2017). It is widely accepted that the majority of molecularly confirmed BrS cases result from a loss-of-function mutation in *SCN5A*, which encodes for a voltage-gated sodium channel subunit (Na_v_1.5). Heterozygous mutations in *SCN5A* are the most commonly diagnosed mutations associated with BrS (Di Resta et al., 2015; Sieira et al., 2016; Curcio et al., 2017) and account for 15–30% of BrS cases (Kapplinger et al., 2010). Nevertheless, a clear molecular confirmation is not achieved in most BrS cases, perhaps due to both locus heterogeneity and genomic imbalances undetectable by NGS methods (Sonoda et al., 2018).

Type 1 neurofibromatosis (NF1) is caused by heterozygous mutations in the *NF1* gene (Gutmann et al., 2017), and it is characterized by pigmentary lesions (café au lait spots, Lisch nodules, freckling) and cutaneous neurofibromas (Friedman, 1993; Montani et al., 2011). Patients affected by NF1 can present with multiple organ involvement and hypertension, and they have a higher risk of developing malignant tumors than others of the same age in the general population (Friedman, 1993; Korf, 1999). The risk of developing gliomas (Friedman, 1993; Matsui et al., 1993) and benign tumors (Matsui et al., 1993; Anik and Abaci, 2014) is even higher than the risk of developing malignant ones, and in some cases, the benign tumors can become malignant (Miettinen et al., 2017). At least half of patients with NF1 also present with learning disabilities (Friedman, 1993). Premature death in NF1 patients is frequently caused by cardiovascular disease, especially since severe complications of NF1 include vasculopathy, hypertension, and congenital heart defects (Friedman et al., 2002).

In the present study, we report for the first time a family in which the nonsense mutation [NM_198056.2:c. 3946C > T (p.Arg1316*)] in the *SCN5A* gene and the frameshift mutation [NM_001042492.2:c.7686delG (p.Ile2563fs*7)] in the *NF1* gene segregate with the clinical phenotypes BrS and NF1, respectively.

## Case Presentation

### Proband (III-3)

**Figure 1:**
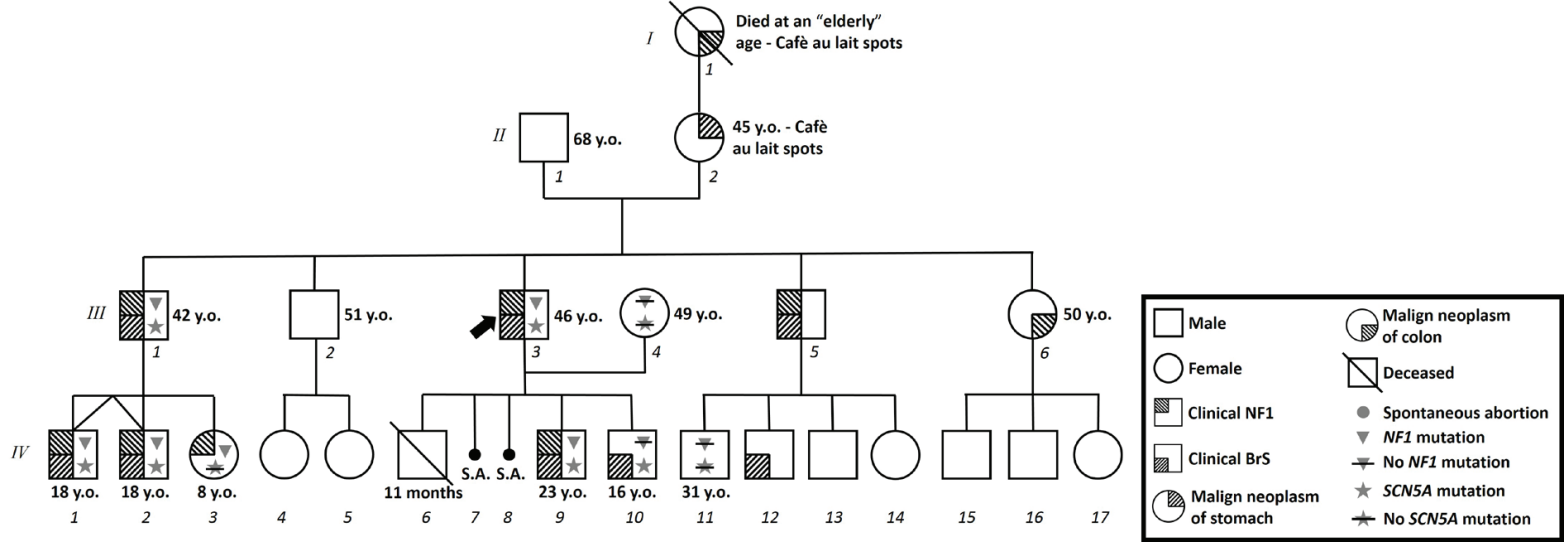
Family pedigree. Proband identified with arrow. Triangle: Molecularly confirmed *NF1* mutation; triangle with slash: genetic test for *NF1* mutation performed but negative; star: molecularly confirmed *SCN5A* mutation; star with slash: genetic test for *SCN5A* mutation performed but negative; y.o.: years old at diagnosis.

Written informed consent of human subjects included in this case series report was obtained for their participation in the study and for publication of this case report. For patients under 18 years old, consent was obtained instead from the parents. The procedures employed were reviewed and approved by the Local Ethics Committee of San Raffaele Hospital, Milan, Italy. The proband is a 46-year-old male of Italian/Caucasian descent who presented with a family history characterized by a strong suspicion of NF1 on the maternal side of the family (Figure 1). In particular, his mother is affected by multiple cafè au lait spots, hyperthyroidism, and stomach malignant neoplasia (at 45 years old). His maternal grandmother had been affected by multiple cafè au lait spots and malignant gut neoplasm (age of onset unknown). A malignant gut neoplasm has been diagnosed recently also in the proband’s sister (50 years old and without other clinical signs of NF1). No consanguinity in the family was reported.

**Figure 2:**
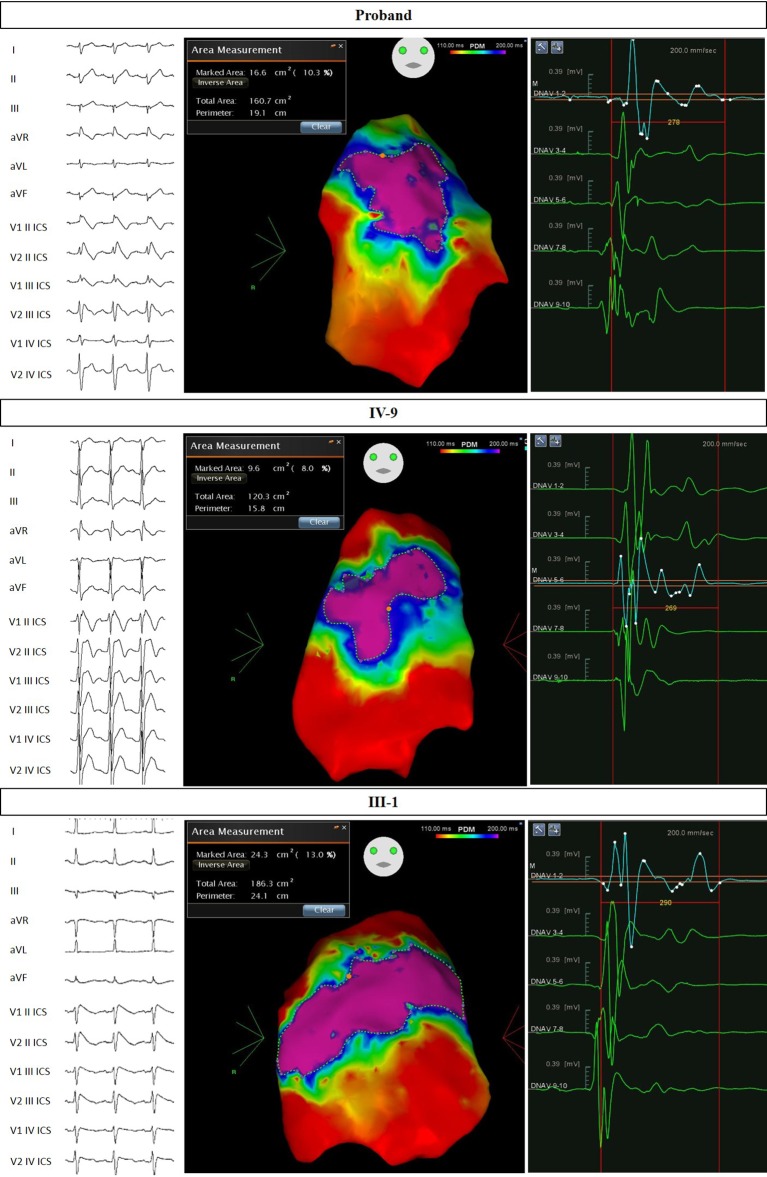
Electrocardiograms and potential duration maps after ajmaline administration and immediately before epicardial catheter ablation. Patients: Proband, IV-9, and III-1.

The proband came to our attention for NF1 genetic counseling. This diagnosis was clinically confirmed based on international criteria (Gutmann et al., 1997). Indeed, our proband was affected by multiple cafè au lait spots, bilateral Lisch nodules, multiple benign skin neoplasms, bilateral axillary freckling, bilateral groin freckling, and a palpable lower left leg subcutaneous mass. We advised the patient of the need for surgical removal of this mass. After a couple of months, the proband underwent surgical removal of four left lower limb masses, all histologically confirmed as “myxoid neurofibroma.” The preoperative ECG for this procedure revealed a type 2 BrS pattern. The patient then returned to us, and we recommended an electrophysiological evaluation. Therefore, the patient underwent an electrophysiological study (EPS), in which the patient tested negative for the inducibility of ventricular arrhythmias. However, the patient tested positive for BrS during a Flecainide test. One year after the first EPS, the proband repeated this procedure and was found to be inducible, and an ICD was subsequently implanted. The patient then underwent successful epicardial ablation of the AS. The potential duration map, which shows the location and extent of the AS after ajmaline administration immediately before epicardial catheter ablation, can be seen in Figure 2.

**Figure 3:**
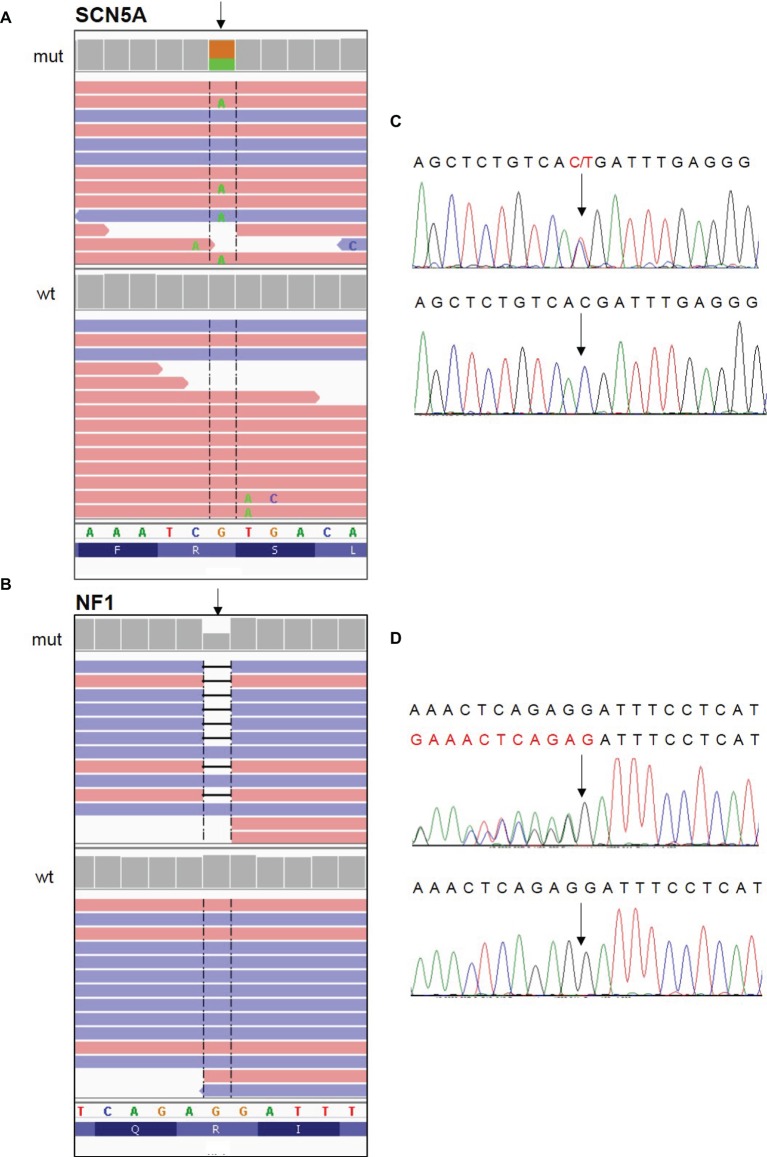
Identification of the c. 3946C > T (p.Arg1316*) nonsense mutation in the SCN5A gene and the c.7686delG (p.Ile2563fs*7) frameshift mutation in the NF1 gene. **(A,B)** NGS paired-end reads loaded in the IGV genome browser. The arrow indicates the position of the single nucleotide variation in SCN5A gene **(A)** and of the deletion in NF1 gene **(B)** in patients (mut) compared to a wt control sample. SCN5A gene is in the reverse orientation on the chromosome. **(C,D)** Sanger sequencing electropherograms confirm the presence of the variants in SCN5A **(C)** and NF1 **(D)** in patients and their absence in the wt control. In the electropherogram of NF1 gene analysis, reverse strand is reported.

The patient underwent genetic testing for both BrS and NF1. Next-generation sequencing was used to analyze genomic DNA extracted from saliva. Results revealed a heterozygous nonsense mutation [c.3946C > T (p.Arg1316*)] in the *SCN5A* gene (LOVD genomic variant #0000406043, https://databases.lovd.nl/shared/variants/0000406043) of unknown parental origin. Sanger sequencing was used to analyze genomic DNA extracted from peripheral blood. Results revealed a heterozygous frameshift mutation [c.7686delG (p.Ile2563fs*7)] in the *NF1* gene (LOVD genomic variant #0000406041, https://databases.lovd.nl/shared/variants/0000406041) of unknown parental origin (Figure 3).

### Older Son (IV-9)

This 23-year-old male patient is the older son of the proband. He was clinically suspected for NF1, presenting with a family history of NF1 (affected father with molecular confirmation), multiple brain hamartoma (likely UBOs), multiple cafè au lait spots, and axillary and groin freckling. This clinical diagnosis was genetically confirmed with the finding of the same mutation in the *NF1* gene found in his father. This patient was inducible during an EPS and diagnosed with BrS after a positive ajmaline test. An ICD was implanted the same day. The patient subsequently underwent successful epicardial catheter ablation of the AS. The potential duration map can be seen in Figure 2. This patient was found to carry the same *SCN5A* mutation found in the proband from genomic DNA extracted from saliva.

### Youngest Son (IV-10)

This 16-year-old male patient is the youngest son of the proband. He presented clinically with delayed speech (after the third year of life), delayed walking (after the fourth year of life), autism spectrum disorder, and one cafè au lait spot. He was tested using genomic DNA extracted from peripheral blood for the *NF1* mutation found in his father. However, he tested negative for this mutation. Taking into consideration mood disturbances, the risk of self-aggressive behavior, and sleep disturbances, the patient was recommended to take valproic acid (750 + 500 mg/day) and risperidone (1.5 mg 2x/day +1.25 mg/day). Due to this therapy, in addition to the family history, the patient performed a 12-lead ECG, which revealed a suspicious pattern suggestive of BrS. An ajmaline test confirmed the diagnosis of BrS. Ajmaline infusion resulted immediately in a type 1 pattern and required isoproterenol infusion to reverse a ventricular arrhythmia that had resulted in a compromise of the hemodynamics. An ICD was implanted the same day. In spite of the BrS diagnosis, the patient has not yet performed an ablation procedure. This patient underwent genetic testing for BrS from genomic DNA extracted from saliva and has been found to carry the same *SCN5A* mutation present in the proband.

### Eldest Brother (III-1)

This 42-year-old male patient is the eldest brother of the proband. He came to our attention with a suspicion of BrS based on both family history and recurrent syncope episodes. An ajmaline test confirmed the diagnosis of BrS, and an EPS was positive for induction of VA. The patient subsequently underwent an ICD implant. The skin examination revealed multiple cafè au lait spots, axillary freckling, and a few cutaneous nodules without histological examination to date. It has been clarified that these clinical elements (together with family history) are sufficient for the clinical diagnosis of NF1, according to international criteria (Gutmann et al., 1997). An ophthalmological evaluation was recommended but has not yet been performed. The patient underwent successful epicardial catheter ablation of the AS. The potential duration map can be seen in Figure 2. This patient was found to have both the *NF1* and *SCN5A* mutations found in the proband from genomic DNA extracted from saliva.

### Nephew (IV-1)

This 18-year-old male patient is the nephew of the proband and son of patient #III-1. At his clinical examination, multiple cafè au lait spots were detected. Taking into consideration the previous diagnosis of NF1 in this patient’s first- and second-degree relatives (father and paternal uncle), NF1 was diagnosed in this patient as well, due to the clinically significant number of cafè au lait spots that totaled more than five and all with a diameter larger than 1.5 cm, according to the international criteria (Williams et al., 2009). It is noteworthy that this patient is also affected by learning disabilities, which is particularly more prevalent in NF1 patients compared with the general population (Torres Nupan et al., 2017).

**Figure 4:**
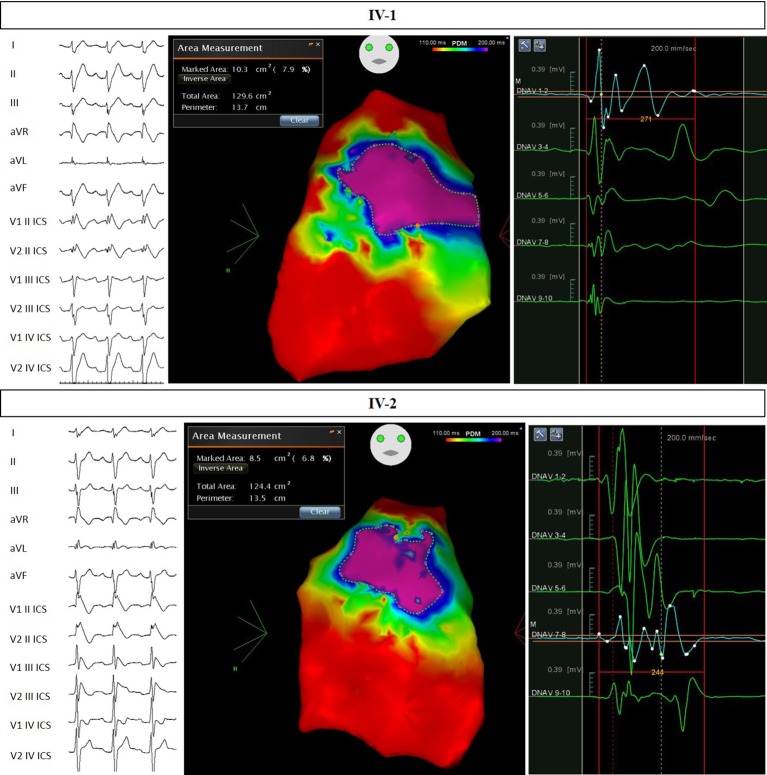
Electrocardiograms and potential duration maps after ajmaline administration and immediately before epicardial catheter ablation. Patients IV-1 and IV-2.

Based on family history, this patient underwent an EPS and ajmaline test, which were both positive for VA inducibility and for the type 1 pattern. For these reasons, he received an ICD. The patient subsequently underwent successful epicardial catheter ablation of the AS. The potential duration map can be seen in Figure 4. This patient was found to have both the *NF1* and *SCN5A* mutations found in other family members from genomic DNA extracted from saliva.

### Nephew (IV-2)

This 18-year-old male patient is the twin brother of patient #IV-1 and thus also the nephew of the proband and son of patient #III-1. At his clinical examination, he showed multiple cafè au lait spots and axillary and groin freckling. Taking into consideration the previous diagnosis of NF1 in this patient’s first- and second-degree relatives, NF1 was diagnosed in this patient as well. Because of the family history, this patient also underwent an EPS and ajmaline test, which were positive for VA inducibility and the type 1 pattern. An ICD was then implanted. The patient subsequently underwent successful epicardial catheter ablation of the AS. The potential duration map can be seen in Figure 4. An echocardiogram demonstrated the presence of a Chiari network (Koz et al., 2008) in the absence of other congenital heart defects. This patient was found to have both the *NF1* and *SCN5A* mutations found in other family members from genomic DNA extracted from saliva.

### Niece (IV-3)

This 8-year-old female was diagnosed with NF1 due to the presence of multiple cafè au lait spots and Lisch nodules, as well as the family history. Due to her age, she has never been tested for BrS to date, but the 12-lead ECG demonstrated a spontaneous normal pattern. This patient is asymptomatic to date, and she was found to be positive for the familiar *NF1* mutation but negative for the familiar *SCN5A* mutation as analyzed from genomic DNA extracted from saliva.

### Nephew (IV-11)

This 31-year-old male patient is another nephew of the proband. While his father (the proband’s brother, III-5) is clinically affected by NF1 and BrS, patient IV-11 did not show any signs of NF1 when clinically examined, and he tested negative for BrS in an ajmaline challenge. This patient was found to be negative for both the *NF1* and *SCN5A* mutations found in other family members from genomic DNA extracted from saliva.

## Discussion

In this case series, we report for the first time a family in which the inherited nonsense mutation [c. 3946C > T (p.Arg1316*)] in the *SCN5A* gene segregates in association with BrS. Moreover, we also report, for the first time, the familial segregation of the novel frameshift mutation [c.7686delG (p.Ile2563fsX40)] in the *NF1* gene and the association of this mutation with NF1. Furthermore, to the best of our knowledge, it is the first time that any family has been reported to have both BrS and NF1. Neurofibromin plays an essential role in cardiac development. Patients with *NF1* mutations exhibit several vascular abnormalities, such as aneurysms or stenosis of the aortic, renal, and mesenteric arteries (Oderich et al., 2007). Tedesco and colleagues reported a higher incidence of heart abnormalities diagnosed by ultrasound in unrelated NF1 patients in sinus rhythm compared to controls (Tedesco et al., 2002). Recently, Incecik and colleagues described a higher incidence of cardiac abnormalities in NF1 pediatric patients, with potentially poor prognoses (Incecik et al., 2015).

NF1 patients present with extreme clinical variability, including even between members of the same family. The majority of NF1 patients with vascular damage are asymptomatic, which can make diagnosis a challenge (Rerat et al., 2015). As a consequence, NF1 vasculopathy is often identified only after autopsy (Hamilton et al., 2001). In cases in which NF1 leads to sudden death, the fatalities usually occur in adulthood and are usually attributed to a central nervous system tumor (Koszyca et al., 1993). However, there are also reports of sudden cardiac death in young children with NF1. Kanter and colleagues described two cases of unrelated children clinically affected by NF1 and by a coronary artery occlusion, causing ventricular fibrillation and sudden death (Kanter et al., 2006). One clinical study investigating the relationship between NF1 and arrhythmias reported fewer cases of bradycardia in the NF1 patient population, suggesting the involvement of the vagus nerve (Malmcrona et al., 1996).

Neurofibromin plays a pivotal role in molecular and cellular pathways, particularly with an inhibitory action on Ras proteins (Upadhyaya, 2010) that are potent factors in triggering cell growth and signaling (Rose et al., 2018). Neurofibromin exists in at least four alternative primary structures. The expression of isoforms 3 and 4 has been discovered in cardiac muscle in rodents (Gutmann et al., 1995). In animal models, neurofibromin is critical for heart development and prevents vascular diseases through Ras downregulation that regulates the development of endocardial cushions, ventricular growth, and fibrosis (Lakkis and Epstein, 1998; Xu et al., 2009). The abolishment of myocardial neurofibromin expression in a knockout mouse model suggested that neurofibromin loss activates the Ras pathway, resulting in progressive cardiac hypertrophy, fibrosis, and cardiac myocyte enlargement (Xu et al., 2009).

It has been widely demonstrated that the protein Na_v_1.5 encoded by the *SCN5A* gene is an essential controller of cardiac excitability, and recent studies underlined that loss of function mutations in *SCN5A* is associated with increased cardiac dimensions and reduced contractility (van Hoorn et al., 2012). Another study by Tedesco and colleagues highlighted that also NF1 patients even without arterial hypertension can show alterations in Doppler tissue imaging (Tedesco et al., 2005). Understanding the clinical significance of individual *SCN5A* mutations is challenging, given the extreme clinical variability seen in patients with *SCN5A* mutations (Kyndt et al., 2001; Six et al., 2008), the variability in the types of mutations and locations within the gene, the number of mutations, genetic heterogeneity, and the fact that some of these variants are found in the general population (Juang et al., 2015). Regardless, nonsense heterozygous mutations in the *SCN5A* gene are generally accepted as causative of BrS (Samani et al., 2009; Gando et al., 2017).

In the present report, two spontaneous abortions occurred in a row, both in the first trimester. Recent literature suggests a genetic study on abortive product after the second consecutive pregnancy loss in the presence of other certain factors that occur at a higher prevalence than 1/100 women (Hyde and Schust, 2015). In such cases, testing for balanced translocation of the chromosomes may be considered (Hyde and Schust, 2015). However, in the present report, the cause of the abortions is unknown.

These data may highlight a close relationship between neurofibromatosis and BrS, because all the members genetically affected were also inducible for VAs during the EPS, which suggests a subset of patients with an aggressive BrS phenotype. Therefore, this may suggest that when neurofibromatosis and BrS are suspected, an extremely careful evaluation of these patients should be performed, as the phenotype manifestation of the combination of these diseases could be life-threatening. Further studies are warranted to investigate these findings in a larger subset of patients.

Taken together these data show how genetic counseling can be useful for a family. The molecular confirmation in asymptomatic members can prompt a clinical examination and preventive interventions. Additionally, affected individuals can be advised about the possibility of avoiding recurrence risk with medically assisted reproduction.

## Concluding Remarks

This study is the first family in which the nonsense mutation [NM_198056.2:c. 3946C > T (p.Arg1316*)] in the *SCN5A* gene and the frameshift mutation [NM_001042492.2:c.7686delG (p.Ile2563fs*7)] in the *NF1* gene segregate with the clinical phenotypes BrS and NF1, respectively. NF1 patients should be routinely checked for cardiac and vascular abnormalities. The co-expression of NF1 and BrS may result in a subset of patients at higher risk of SCD who require the appropriate electrophysiological evaluation. The importance of genetic testing should be emphasized to identify family members who require clinical examinations and preventive interventions, as well as to advise about the possibility of avoiding recurrence risk with medically assisted reproduction.

## Data Availability

The datasets generated for this study can be found in LOVD, LOVD genomic variant #0000406043 and #0000406041.

## Author Contributions

EM, MM, GC, GV, MC, VM, LG, FG, AP, MS, SC, VB, AG, SD, CR, SB, MF, VS, LA, and CP collected/analyzed the data. EM and MM wrote the manuscript. All authors interpreted the results, critically reviewed/edited the manuscript, and approved the final version.

### Conflict of Interest Statement

The authors declare that the research was conducted in the absence of any commercial or financial relationships that could be construed as a potential conflict of interest.
